# Prevalence of frequent mental distress and associated factors among stroke survivors

**DOI:** 10.1371/journal.pone.0361900

**Published:** 2026-11-11

**Authors:** Hayley Clark, Ajith Kumar Vemuri, Alireza Vafaei Sadr, Vida Abedi, Ramin Zand

**Affiliations:** 1 Department of Neurology, College of Medicine, The Pennsylvania State University, Hershey, Pennsylvania, United States of America; 2 Department of Public Health Sciences, College of Medicine, Pennsylvania State University, Hershey, Pennsylvania, United States of America; SDM College of Medical Sciences and Hospital, INDIA

## Abstract

Stroke survivors face considerable risk for mental health complications including depression, anxiety, and mental distress. Despite evidence linking mental health to stroke, frequent mental distress (FMD), a broader public health marker of mental health burden, remains underexamined in stroke populations. This study aims to find the prevalence and associated factors of FMD among U.S. stroke survivors. Analysis was expanded to compare FMD with individuals who have a history of myocardial infarction (MI) and healthy adults. We conducted a cross-sectional analysis using Behavioral Risk Factor Surveillance System data from 2016 to 2023. FMD was defined in two different ways, with the standard threshold of ≥14 days of poor mental health in the past 30 days, and an alternative ≥6-day threshold. Weighted prevalence estimates and multivariate logistic regressions examined associations between social and demographic factors and FMD. Comparative analyses assessed differences in FMD between stroke and MI survivors with the healthy adult population as reference. FMD was most prevalent among stroke survivors who were younger adults (18–64) at 32.5%, females at 26.0%, and Hispanics at 28.7%. Geographically, the highest FMD rates occurred in West Virginia (29.0%) and Tennessee (27.6%), concentrated in the Stroke Belt and Appalachian regions. Stroke survivors had significantly greater odds of experiencing FMD than healthy adults in both unadjusted (4.36) and adjusted regression models (1.96). MI survivors also had greater odds of FMD compared to healthy adults (3.02 unadjusted, 1.69 adjusted), but FMD was less likely when compared to stroke survivors. Our findings suggest that FMD is more prevalent in younger adults, females, Hispanic individuals, and those residing in the Stroke belt and Appalachian states. FMD is also more prevalent among stroke survivors compared to individuals with MI or healthy adults. Future research is necessary for targeted mental health interventions in these specific subgroups.

## Introduction

In 2022, there were an estimated 59.3 million adults in the United States with a mental illness, representing 23.1% of all U.S. adults [1]. Stroke survivors experience an even greater burden of mental health as over one third develop a clinically significant mental disorder [2]. Although this prevalence is significant, it misses out on a large population of people who may not be diagnosed with a mental disorder or are just below the threshold of diagnosis, showing a need to explore a more encompassing definition of mental health [3,4].

Various studies have looked at the prevalence of mental illnesses including depression, anxiety, and mental distress among stroke survivors; however, these studies are limited. The INTERSTROKE case-control study found those with depression had a 1.35 increased prevalence of having a stroke, but the findings relied on hospital-based samples with limited generalizability [5]. Two meta-analyses found that 29% of stroke survivors experience post-stroke depression and that having an anxiety disorder increases stroke prevalence by 24%, while a narrative review reported similar findings of post-stroke depression and noted that post-stroke anxiety persists in over 20% of patients one year after stroke [6–8]. These studies are limited by methodological heterogeneity across studies, reliance on a single data extractor, and use of older data ranging from 1977 to 2016 across all three reviews. One cross-sectional study examined associations between frequent mental distress (FMD), sociodemographic factors, and chronic conditions, finding a significant link between FMD and stroke [9]. However, it did not specifically analyze the sociodemographic characteristics of stroke survivors or explore alternative FMD definitions through sensitivity analyses.

Building on these limitations in the existing literature, the present study aims to provide a more current and comprehensive assessment of mental health among stroke survivors. The objectives of this study are: 1) to estimate the prevalence of FMD among U.S. adults with a history of stroke; 2) to identify sociodemographic and geographic factors associated with FMD of stroke survivors; and 3) to conduct a comparative analysis of FMD among stroke survivors versus those with a history of myocardial infarction (MI) and healthy adults. We hypothesize that sociodemographic and geographic factors are associated with FMD among stroke survivors.

## Methods

### Design

In this study, we analyzed the data from the Behavioral Risk Factor Surveillance System (BRFSS) surveys conducted from 2016 to 2023 [10–17]. The BRFSS is an annual, cross-sectional telephone survey administered by Centers for Disease Control and Prevention (CDC). It collects data from randomly selected adults in all 50 states, the District of Columbia, and U.S. territories, using both landline and cellular telephone interviews. To avoid selection bias, the BRFSS randomly selects telephone numbers and makes calls 7 days a week throughout the day. The response rate of BRFSS for the most recent year (2023) was 44.7%, and BRFSS provides sample weights to account for nonresponse rates while ensuring the resulting weights represent the U.S. population [18]. We accessed this data on June 24^th^, 2025.We adhered to the STROBE guidelines to report our findings [19]. Informed consent and ethics committee approval are not needed for this study as the data is public and de-identified.

### Eligibility criteria

The BRFSS survey included household adults aged 18 years or older. Every year, >400,000 adults are interviewed. Our analysis excluded respondents who did not report information about stroke and mental health. Additionally, for our regression analyses, we excluded individuals with missing data in sociodemographic factors (S1 Fig and S2 Table).

For our comparative analysis the myocardial infarction (MI) population included all MI survivors with the required mental health and demographic data. The healthy adult population excluded all respondents who reported having the following conditions: stroke, MI, heart disease (including coronary heart disease and angina), asthma, cancer, chronic obstructive pulmonary disease (COPD), arthritis, major depression, kidney disease, and diabetes. Stroke and MI status were determined based on self-report, which has been shown to be valid and reliable within the BRFSS dataset [20].

### Variable definitions

This study assessed several sociodemographic variables, including age, race, sex, body mass index (BMI), annual household income, health insurance status, education status, stroke belt residence, and rural status. Age was self-reported and categorized into two groups: 18–64 years and ≥65 years. Race was classified into five categories: non-Hispanic White, non-Hispanic Black, non-Hispanic other races, non-Hispanic multiracial, and Hispanic. Sex was recorded as either male or female. BMI was a calculated variable based on the respondent's height and weight that was categorized as underweight (<18.5 kg/m^2^), normal weight (18.5–24.9 kg/m^2^), overweight (25.0–29.9 kg/m^2^), or obese (≥30.0 kg/m^2^). Annual household income was grouped into seven categories: less than $15,000; $15,000 to less than $25,000; $25,000 to less than $35,000; $35,000 to less than $50,000; $50,000 to less than $100,000; $100,000 to less than $200,000; and $200,000 or more. Health insurance status included any form of public or private coverage at the time of the survey. Education status was categorized as: did not graduate high school, graduated high school, attended college or technical school, and graduated from college or technical school. Stroke belt residence was defined as living in one of eight U.S. states with historically elevated stroke mortality rates: Alabama, Arkansas, Georgia, Louisiana, Mississippi, North Carolina, South Carolina, and Tennessee. Rural status was defined using the Metropolitan Status Code with respondents residing in counties not designated as part of a Metropolitan Statistical Area (MSA) being classified as rural, while those residing within an MSA were classified as urban. All variables were derived from self-reported responses in the BRFSS dataset. All variables are defined in S1 Table.

### Outcome measures

We define the outcome variable, frequent mental distress (FMD), based on the BRFSS question: “Now thinking about your mental health, which includes stress, depression, and problems with emotions, for how many days during the past 30 days was your mental health not good?”. Respondents who reported 14 or more days of poor mental health in the past 30 days were classified as experiencing FMD. This definition was created by the Centers for Disease Control and Prevention (CDC), as it aligns with the two-week diagnostic criteria for depressive episodes in the ICD-11 and is widely used in population health research [21–27]. As a secondary analysis we also explored a 6-day threshold for FMD as proposed and validated by Bossarte et al [22]., which may increase sensitivity to more transient or moderate distress.

### Statistical analysis

Data from the 2016–2023 BRFSS annual surveys were merged to create a pooled dataset. To calculate population-weighted frequencies and proportions, we used survey-specific R functions that accounted for the complex sampling design, including weights, strata, and primary sampling units. For our primary analysis, the Rao–Scott chi-squared test was used to assess differences in the prevalence of FMD across demographic strata (e.g., male vs. female).

We report the sociodemographic characteristics of the study population and the percentage of respondents who experienced FMD across all demographic variables. To examine associations between sociodemographic factors and FMD, we conducted a secondary analysis of both single-variate (unadjusted) and multi-variate (adjusted) regression analyses. Adjusted models included age, race, sex, BMI, income, health insurance status, and education status as covariates. For each factor, we calculated unadjusted and adjusted odds ratios with corresponding confidence intervals. Additionally, we performed regression analyses to compare the odds of FMD among stroke survivors and myocardial infarction (MI) survivors, using healthy adults as the reference group. The same set of covariates was used for adjustment in these models. We also analyzed the distribution of FMD in stroke survivors in the United States geographically. Lastly, we looked at the temporal trends of FMD among stroke survivors from the years 2016–2023. Statistical analysis was performed with R 4.5.1. Forest plots for odds ratios were generated using the ggplot package in R. State-wise FMD percentages were plotted using Data Wrapper. *p* < 0.05 was considered statistically significant.

## Results

### General characteristics of the studied population

We analyzed BRFSS survey data which included adults 18 years or older. After excluding respondents with missing mental health data, a complete analysis was done on 143,370 stroke survivors. Table 1 includes the general characteristics of the study population. Among the stroke survivors, 49.6% were aged 18–64 and 48.2% were male. Approximately 64.5% identified as white only, 16.5% as black only, and 11.7% as Hispanic. For BMI, 37.7% were categorized into the obese range, 34.4% were overweight, 25.8% were normal weight, and 2.1% were underweight. A total of 25.8% of individuals reported residing in the Stroke Belt and 6.6% of stroke survivors did not have health insurance. For education, 21.0% did not graduate high school, 30.7% graduated high school, 31.0% attended college or technical school, and 17.3% graduated from college or technical school.

**Table 1:** General characteristics of stroke survivors.

Variable	Level	Unweighted Count (n)	Weighted Percent (%)
Age	Age 18 to 64	52,559	49.63
	Age 65 or older	89,177	50.37
Race	White only, Non-Hispanic	106,418	64.53
	Black only, Non-Hispanic	15,507	16.54
	Other race only, Non- Hispanic	6,822	5.03
	Multiracial, Non-Hispanic	3,769	2.22
	Hispanic	7,511	11.67
Sex	Male	64,953	48.23
	Female	78,441	51.77
BMI	Underweight	2,861	2.06
	Normal Weight	35,616	25.81
	Overweight	46,300	34.42
	Obese	48,662	37.71
Income	Less than $15,000	20,175	19.10
	$15,000 to < $25,000	27,156	23.84
	$25,000 to < $35,000	16,613	14.05
	$35,000 to < $50,000	16,020	13.40
	$50,000 to < $100,000	29,194	24.79
	$100,000 to < $200,000	3,950	3.74
	$200,000 or more	982	1.08
Health Insurance	Insured	134,761	93.40
	Uninsured	6,141	6.60
Education Status	Did not graduate High School	16,824	21.03
	Graduated High School	45,667	30.73
	Attended College or Technical School	42,468	30.95
	Graduated from College or Technical School	37,946	17.29
Stroke Belt Residence	Non-stroke belt	112,526	74.24
	Stroke belt	30,944	25.76
Rural Status	Non-rural	42,034	77.73
	Rural	23,473	22.27

### Prevalence of FMD days among stroke survivors in the United States

For our primary analysis, the prevalence of FMD, defined as 14 or more days of “not good” mental health in the past 30 days, varied significantly across sociodemographic factors (Table 2). Among the stroke population, younger individuals (ages 18–64) had higher FMD compared to those aged 65 and older (32.5% vs. 14.6%; p < 0.00001). Females exhibited greater FMD at 26.0% compared to males at 20.6%. FMD prevalence showed a strong inverse relationship with household income. Stroke survivors earning less than $15,000 annually had the highest rate of FMD (37.1%), while those earning $200,000 or more reported the lowest (12.7%). By race and ethnicity, Hispanic individuals reported higher FMD (28.7%), when compared to White only (21.9%) and Black only (23.4%) individuals. Educational status showed a similar trend to income, with FMD rates decreasing as education level increased. Respondents who did not graduate high school had a 30.6% prevalence of FMD, while college or technical school graduates reported significantly lower rates at 14.9%. Stroke Belt residents had a higher prevalence of frequent distress (25.5%) compared to those living outside the region (22.7%).

**Table 2:** Demographic factors and days with not good mental health percentage among stroke survivors with FMD defined as ≥14 days of poor mental health in the past 30 days.

Factor	N (Unweighted)	Zero Days with Not Good Mental Health (95% CI) (Weighted)	1 to 13 Days with Not Good Mental Health (95% CI) (Weighted)	14 or More Days with Not Good Mental Health (95% CI) (Weighted)	*p*-Value
**Age**					< 0.0001
Age 18 to 64	52559	43.3 (42.4-44.2)	24.2 (23.4-25.0)	32.5 (31.7-33.4)	
Age 65 or older	89177	67.6 (66.9-68.3)	17.8 (17.2-18.4)	14.6 (14.0-15.1)	
**Race**					< 0.0001
White only, Non-Hispanic	106418	57.9 (57.3-58.6)	20.1 (19.6-20.7)	21.9 (21.4-22.5)	
Black only, Non-Hispanic	15507	53.5 (51.9-55.1)	23.1 (21.6-24.5)	23.4 (22.1-24.7)	
Other race only, Non-Hispanic	6822	52.0 (48.6-55.5)	23.5 (20.5-26.5)	24.4 (21.8-27.0)	
Multiracial, Non-Hispanic	3769	45.8 (41.9-49.7)	23.9 (20.8-27.0)	30.3 (27.0-33.6)	
Hispanic	7511	50.2 (47.8-52.7)	21.0 (19.1-23.0)	28.7 (26.5-31.0)	
**Sex**					< 0.0001
Male	64953	60.9 (60.1-61.8)	18.4 (17.8-19.1)	20.6 (19.9-21.3)	
Female	78441	50.8 (50.0-51.6)	23.2 (22.5-23.9)	26.0 (25.2-26.7)	
**BMI**					< 0.0001
Underweight	2861	47.2 (43.2-51.1)	20.0 (16.9-23.0)	32.9 (29.0-36.8)	
Normal Weight	35616	56.7 (55.5-57.9)	21.3 (20.3-22.3)	22.0 (21.0-23.0)	
Overweight	46300	59.7 (58.7-60.7)	19.9 (19.1-20.7)	20.4 (19.6-21.2)	
Obese	48662	51.5 (50.5-52.5)	22.0 (21.2-22.9)	26.5 (25.6-27.4)	
**Income**					< 0.0001
Less than$15,000	20175	40.8 (39.3-42.3)	22.1 (20.6-23.7)	37.1 (35.5-38.6)	
$15,000 to < $25,000	27156	51.1 (49.8-52.4)	21.1 (20.0-22.1)	27.8 (26.6-29.0)	
$25,000 to < $35,000	16613	55.6 (54.0-57.3)	21.6 (20.2-22.9)	22.8 (21.4-24.2)	
$35,000 to < $50,000	16020	60.7 (58.9-62.4)	21.3 (19.8-22.7)	18.1 (16.7-19.4)	
$50,000 to < $100,000	29194	62.8 (61.4-64.1)	21.8 (20.7-22.9)	15.4 (14.4-16.4)	
$100,000 to < $200,000	3950	61.7 (58.1-65.3)	25.0 (21.9-28.2)	13.2 (10.6-15.8)	
$200,000 or more	982	58.8 (52.3-65.3)	28.5 (22.1-34.9)	12.7 (8.7-16.7)	
Health Insurance					< 0.0001
Insured	134761	56.8 (56.2-57.4)	20.8 (20.3-21.3)	22.5 (21.9-23.0)	
Uninsured	6141	41.1 (38.4-43.8)	23.6 (20.9-26.3)	35.4 (32.7-38.0)	
Education Status					< 0.0001
Did not graduate high school	16824	50.0 (48.4-51.5)	19.4 (18.1-20.7)	30.6 (29.2-32.0)	
Graduated high school	45667	56.0 (54.9-57.0)	20.2 (19.3-21.0)	23.9 (23.0-24.8)	
Attended college or technical school	42468	55.7 (54.7-56.8)	21.5 (20.6-22.3)	22.8 (21.9-23.7)	
Graduated from college or technical school	37946	61.8 (60.7-62.9)	23.3 (22.2-24.3)	14.9 (14.1-15.7)	
**Stroke Belt Residence**					< 0.0001
Non-stroke	112526	56.0 (55.2-56.7)	21.3 (20.7-22.0)	22.7 (22.1-23.3)	
Stroke Belt	30944	54.8 (53.9-55.7)	19.7 (19.0-20.5)	25.5 (24.7-26.3)	
**Rural Status**					0.2
Non-rural	42034	62.2 (61.1-63.3)	19.3 (18.3-20.2)	18.5 (17.6-19.4)	
Rural	23473	60.9 (59.2-62.6)	19.0 (17.6-20.5)	20.1 (18.7-21.4)	

We also examined the prevalence of FMD using an alternative threshold of 6 or more days of poor mental health in the past 30 days (S3 Table). The direction of FMD across sociodemographic factors remained consistent for both the 6-day and 14-day definitions. Younger age, female sex, Hispanic ethnicity, lower household income, lower educational status, lack of health insurance, and residence within the Stroke Belt continued to be associated with elevated FMD prevalence.

### Factors associated with FMD among stroke survivors based on regression analysis

Multivariate regression analyses identified several sociodemographic factors significantly associated with frequent mental distress among stroke survivors using the 14-day definition of FMD (Table 3). We excluded respondents with missing data in mental health and sociodemographic factors with a total of 105,689 stroke survivors. After adjusting for covariates, individuals aged 65 years or older had lower odds of FMD compared to those under the age of 65 (AOR: 0.37 [95% CI: 0.35–0.4]) and females had higher odds of FMD relative to males (AOR: 1.38 [95% CI: 1.29–1.48]). Among racial and ethnic groups, Hispanic stroke survivors had greater odds of FMD compared to White respondents in unadjusted analyses (OR: 1.37 [95% CI: 1.2–1.56]), but this association was not sustained after adjustment. In contrast, Black stroke survivors exhibited lower odds of FMD relative to White individuals (AOR: 0.73 [95% CI: 0.66–0.81]). Income was inversely associated with FMD as stroke survivors earning $200,000 or more annually had reduced odds (AOR: 0.22 [95% CI: 0.14–0.35]) compared to those earning less than $15,000. Education followed a similar pattern, with college graduates showing significantly lower odds of FMD (AOR: 0.67 [95% CI: 0.59–0.76]) relative to those without a high school diploma.

**Table 3:** Odds ratio of stroke survivor FMD, defined as ≥14 days of poor mental health in the past 30 days, in the United States.

Variable (Reference)	Unadjusted Odds Ratio (95% CI)	*p*-Value	Adjusted Odds Ratio (95% CI)	*p*-Value
**Age**				
Less than 65 (reference)	–	–	–	–
65 and above	0.36 (0.33 - 0.38)	< 0.0001	0.37 (0.35 - 0.4)	< 0.0001
**Race**				
White (reference)	–	–	–	–
Black only, non-Hispanic	1.06 (0.96 - 1.16)	0.2401	0.73 (0.66 - 0.81)	< 0.0001
Other races only, non-Hispanic	1.1 (0.93 - 1.3)	0.2568	0.93 (0.78 - 1.1)	0.3791
Multiracial, non-Hispanic	1.53 (1.29 - 1.83)	< 0.0001	1.22 (1.01 - 1.47)	< 0.05
Hispanic	1.37 (1.2 - 1.56)	< 0.0001	0.88 (0.76 - 1.02)	0.0811
**Sex**				
Male (reference)	–	–	–	–
Female	1.44 (1.35 - 1.53)	< 0.0001	1.38 (1.29 - 1.48)	< 0.0001
**BMI**				
Underweight (reference)	–	–	–	–
Normal Weight	0.51 (0.42 - 0.63)	< 0.0001	0.61 (0.49 - 0.77)	< 0.0001
Overweight	0.48 (0.39 - 0.59)	< 0.0001	0.61 (0.49 - 0.76)	< 0.0001
Obese	0.65 (0.53 - 0.8)	< 0.05	0.69 (0.55 - 0.86)	< 0.05
**Income**				
Less than $15,000 (reference)	–	–	–	–
$15,000 to <$25,000	0.63 (0.58 - 0.69)	< 0.0001	0.73 (0.66 - 0.8)	< 0.0001
$25,000 to <$35,000	0.49 (0.44 - 0.55)	< 0.0001	0.6 (0.53 - 0.67)	< 0.0001
$35,000 to <$50,000	0.35 (0.31 - 0.39)	< 0.0001	0.44 (0.39 - 0.5)	< 0.0001
$50,000 to <$100,000	0.29 (0.26 - 0.33)	< 0.0001	0.36 (0.32 - 0.41)	< 0.0001
$100,000 to <$200,000	0.24 (0.19 - 0.31)	< 0.0001	0.29 (0.22 - 0.37)	< 0.0001
$200,000 or more	0.18 (0.12 - 0.29)	< 0.0001	0.22 (0.14 - 0.35)	< 0.0001
**Health Insurance**				
Insured (reference)	–	–	–	–
Uninsured	1.99 (1.73 - 2.28)	< 0.0001	1.21 (1.05 - 1.41)	< 0.05
**Education Status**				
Did not graduate high school (reference)	–	–	–	–
Graduated high school	0.69 (0.63 - 0.76)	< 0.0001	0.83 (0.75 - 0.92)	< 0.05
Attended college or technical school	0.66 (0.6 - 0.73)	< 0.0001	0.89 (0.8 - 0.99)	< 0.05
Graduated from college or technical school	0.38 (0.34 - 0.42)	< 0.0001	0.67 (0.59 - 0.76)	< 0.0001

Adjusted for sociodemographic factors (age, race, sex, BMI, income, health insurance, education status).

To further evaluate the sensitivity of sociodemographic associations with frequent mental distress, we conducted a second multivariate regression using a 6-day definition of FMD (S4 Table). The direction and strength of associations were similar under the 14-day model. Older age, male sex, White race, higher income, higher education status, normal or overweight BMI, and health insurance coverage remained protective.

### Associations of FMD among stroke survivors, MI survivors, and the healthy population

Multivariate regression analyses using the 14-day definition of FMD revealed significantly higher odds of FMD among individuals with a history of stroke or MI compared to healthy adults (Fig 1). In the unadjusted model, stroke survivors had an odds ratio of 4.36 and MI survivors 3.02. After controlling for age, sex, race, BMI, income, health insurance, education status, and depression, the pattern remained consistent with adjusted odds ratios of 1.96 for stroke and 1.69 for MI.

**Fig 1:**
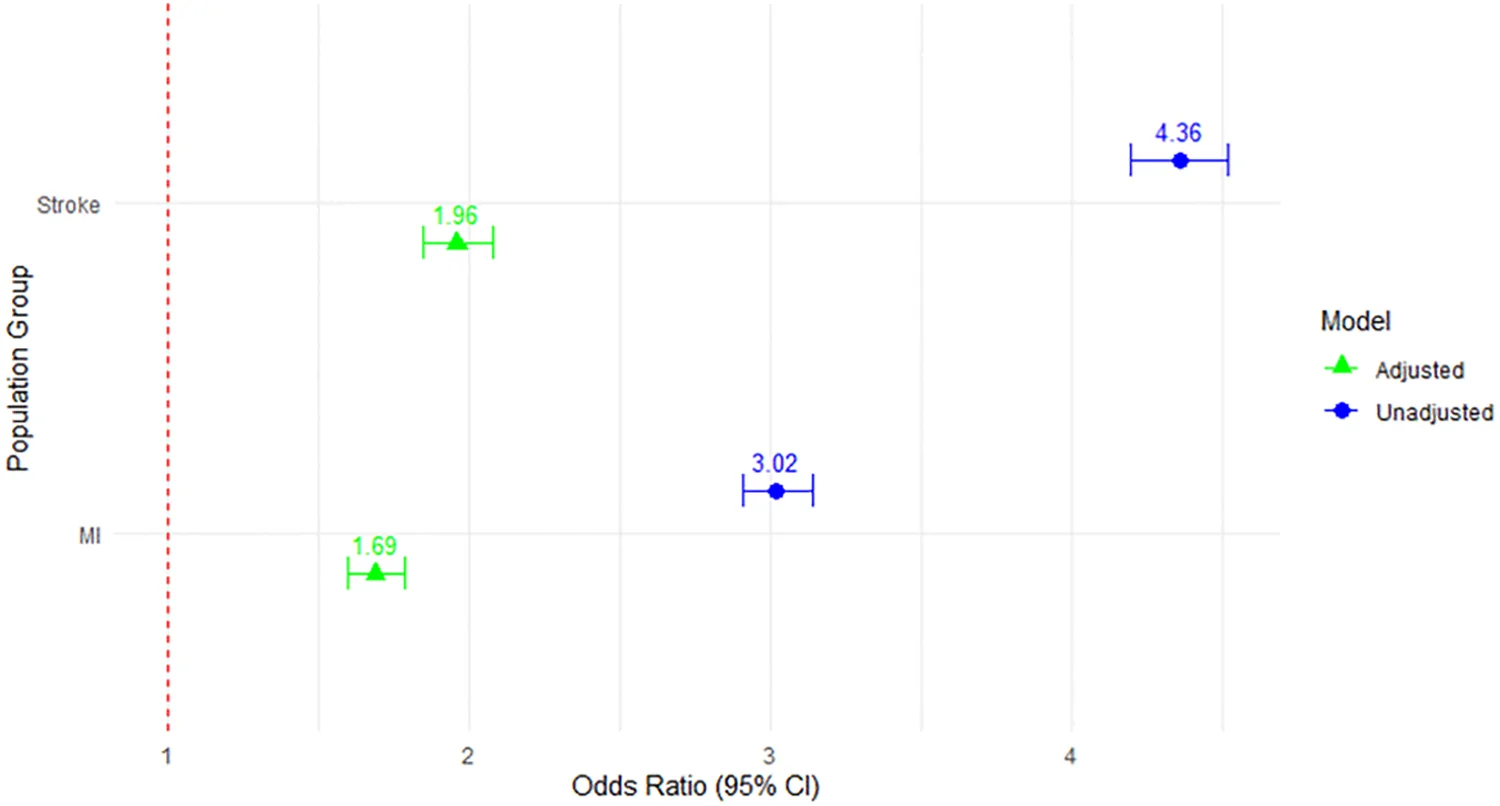
Unadjusted and adjusted odds ratios of FMD, defined as ≥14 days of poor mental health in the past 30 days, among stroke and MI survivors compared to the healthy adult population.

Using the 6-day FMD definition, regression analysis produced comparable findings (S2 Fig). Stroke and MI survivors demonstrated higher odds, with adjusted ratios of 1.73 and 1.45, respectively.

### Prevalence of FMD geographically

Considerable variability in FMD was found geographically (Fig 2). FMD varied between states with North Dakota at 12.5% and West Virginia at 29.0%. Within the Stroke Belt, FMD prevalence among stroke survivors ranges from 20.6% in South Carolina to 27.6% in Tennessee, well above the lower bounds. The rest of the Stroke Belt states sit above 22.0%, continuing the trend of stroke survivors in the Stroke Belt having higher FMD. Outside the Stroke Belt, West Virginia (29.0%) shows the highest overall prevalence, surpassing even the Stroke Belt states. Kentucky (26.4%) also ranks among the most affected demonstrating higher burden of mental health in the broader Appalachian and southeast region. In contrast, states like North Dakota (12.5%), South Dakota (15.0%), Nebraska (16.6%), and Minnesota (16.9%) report markedly lower rates.

**Fig 2:**
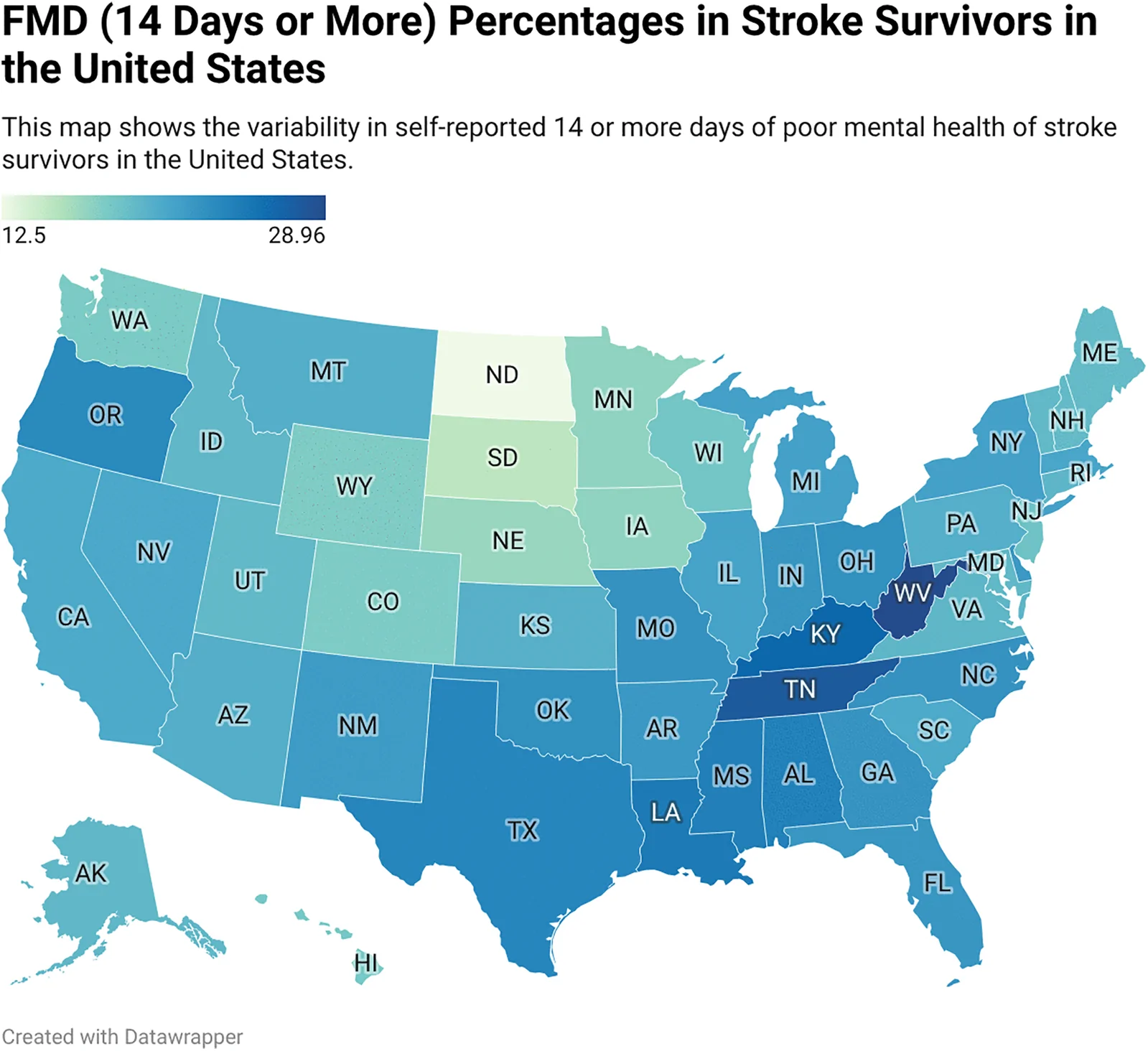
FMD (14 or more days) percentages in stroke survivors in the United States. AL: Alabama, AK: Alaska, AZ: Arizona, AR: Arkansas, CA: California, CO: Colorado, CT: Connecticut, DE: Delaware, DC: District of Columbia, FL: Florida, GA: Georgia, HI: Hawaii, ID: Idaho, IL: Illinois, IN: Indiana, IA: Iowa, KS: Kansas, KY: Kentucky, LA: Louisiana, ME: Maine, MD: Maryland, MA: Massachusetts, MI: Michigan, MN: Minnesota, MS: Mississippi, MO: Missouri, MT: Montana, NE: Nebraska, NV: Nevada, NH: New Hampshire, NJ: New Jersey, NM: New Mexico, NY: New York, NC: North Carolina, ND: North Dakota, OH: Ohio, OK: Oklahoma, OR: Oregon, PA: Pennsylvania, RI: Rhode Island, SC: South Carolina, SD: South Dakota, TN: Tennessee, TX: Texas. UT: Utah. VT: Vermont, VA: Virginia, WA: Washington, WV: West Virginia, WI: Wisconsin, WY: Wyoming.

Geographic analysis using the 6-day FMD definition produced results consistent with the 14-day findings (S3 Fig). West Virginia continued to report the highest prevalence of FMD at 36.0%, while North Dakota had the lowest at 17.1%. Within the Stroke Belt, FMD prevalence ranged from 27.4% in South Carolina to 34.8% in Tennessee. The other Stroke Belt states reported significant rates exceeding 27.5%. A broader regional pattern was also comparable to the 14-day threshold, with the southeastern U.S. consistently demonstrating higher levels of FMD compared to northern and western states.

## Discussion

Our study used 8 years of nationally representative BRFSS data to examine FMD among U.S. stroke survivors, revealing several key findings. First, FMD was highly prevalent among younger adults, females, Hispanic individuals, those with lower income, lower education status, and the uninsured. Second, stroke survivors residing in the Stroke Belt and Appalachian states exhibited consistently higher rates of FMD than those in other U.S. regions. Third, the prevalence of FMD was significantly higher among stroke survivors compared to healthy adults and was higher than those with a history of MI, regardless of the FMD threshold or adjustment for covariates.

The prevalence of FMD among the identified sociodemographic groups remained consistent across both the ≥ 14-day and ≥6-day definitions of FMD. Our results reinforce prior studies linking younger age, female sex, and social disadvantage to increased risk of post-stroke depression and anxiety [8,28,29]. Notably, our results identified Hispanic individuals experiencing a disproportionately high prevalence of FMD among stroke survivors compared to Black individuals when prior research has shown the highest rates of FMD were among Black individuals in the general population, suggesting that the distribution of FMD may differ based on chronic health conditions [24,30].

Although our population is of adults in the U.S., our findings also align with global research on post-stroke mental health. Studies conducted in England and Nigeria found that the prevalence of post-stroke anxiety and depression, respectively, were similar to the FMD rates observed in our U.S. sample [31,32]. These international studies similarly reported that younger adult females were at greatest risk, suggesting that the demographic patterns of stroke survivors’ mental health burden may be consistent globally.

Geographic variation in FMD among stroke survivors was also substantial. State-level FMD prevalence ranged from 12.5% in North Dakota to 29.0% in West Virginia, with consistently elevated rates observed in the southeastern U.S., including Stroke Belt and Appalachian states. This is a new contribution to the literature as no previous studies have assessed the prevalence of mental health of stroke survivors by state.

Our comparative analysis found that stroke survivors consistently reported higher levels of FMD than those with a history of MI. This difference remained significant after adjusting for sociodemographic factors and using both definitions of FMD. To our knowledge, no prior studies have directly compared FMD between these two cardiovascular conditions. While one study found a 2.5-fold increase in psychological distress among individuals with MI compared to those without [33], our findings suggest that stroke survivors experience even greater mental health burden. This may be due to stroke’s unique neurological effects, including cognitive impairment driven by white matter alterations, disability, and emotional dysregulation, which can more profoundly affect quality of life than MI in many cases [34–36].The implications of these findings underscore the importance of integrating mental health support into both public health strategies and routine stroke care. The elevated FMD among specific sociodemographic groups point to the need for targeted mental health care among stroke patients, and the geographic trends of FMD highlight the importance of regional interventions. Additionally, the greater FMD in stroke compared to MI suggests that stroke- specific mental health sequelae deserve more attention in research. Future research should explore incorporating mental health care as a core component of pre-stroke intervention and post-stroke rehabilitation, especially in identified sociodemographic groups, to see if it has an impact on reducing incidence or improving long-term outcomes.

This study has several strengths, including its large, nationally representative sample, consistent use of validated FMD definitions, and a multi-faceted analytic approach. However, limitations should be noted. First, the BRFSS does not include data on institutionalized stroke survivors as respondents are volunteers, but the data is weighted to account for noncoverage and nonresponse. Second, both stroke history and mental health symptoms were self-reported, which may lead to underreporting particularly among older adults, stroke survivors with more severe disabilities like cognitive impairments and aphasia, or individuals affected by stigma [8,37]. Also, some questions within the dataset were not asked by every person taking the survey, making additional factors, like cognitive impairment of the study population, not possible to analyze. Furthermore, our use of FMD is supported by evidence validating both the 14- and 6-day thresholds as reliable indicators of generalized distress linked to clinical depression and serious mental illness [22]; however, as a non-diagnostic, single-item measure, FMD cannot capture specific disorders, symptom severity, or suicidality, limiting assessment precision in this population. Additional clinical data show that very old stroke patients (≥85 years) have distinct demographic and vascular risk profiles that differ from younger stroke populations [38]. Because BRFSS lacks clinical detail and cannot isolate this subgroup, our study could not evaluate whether FMD patterns vary in this older population. Likewise, BRFSS does not capture sleep quality or sleep‑related breathing disorders, despite evidence that poor sleep and obstructive sleep apnea are associated with worse stroke outcomes [39]. Lastly, the BRFSS lacks data on respondents’ use and adherence of anti-depressant medications which may be a significant confounder.

## Conclusion

Our study demonstrates that frequent mental distress (FMD) is significantly more prevalent among stroke survivors compared to individuals with myocardial infarction (MI) or those without major health conditions. Higher rates of FMD were consistently observed among females, Hispanic individuals, those with lower income, lower education status, uninsured, and stroke survivors residing in the Stroke Belt and Appalachian states. FMD among U.S. stroke survivors are associated with notable sociodemographic and geographic factors, and higher FMD compared to MI survivors. The persistence of these disparities across multiple thresholds demonstrate the complexity and urgency of addressing mental health within stroke populations. Together, these results highlight the need to integrate targeted mental health support into post‑stroke care. Future research that incorporates more detailed clinical data, such as isolated age‑specific analyses, sleep quality, and antidepressant use, will help address current gaps in knowledge to refine our understanding of the mental health burden faced by stroke survivors.

## Supporting information

S1 Fig Inclusion/Exclusion criteria for the study. (DOCX)

S2 Fig Adjusted and unadjusted odds ratios of FMD, defined as ≥6 days of poor mental health in the past 30 days, among stroke and MI survivors compared to the healthy adult population. (DOCX)

S3 Fig FMD (6 or more days) percentages in stroke survivors in the United States. AL: Alabama, AK: Alaska, AZ: Arizona, AR: Arkansas, CA: California, CO: Colorado, CT: Connecticut, DE: Delaware, DC: District of Columbia, FL: Florida, GA: Georgia, HI: Hawaii, ID: Idaho, IL: Illinois, IN: Indiana, IA: Iowa, KS: Kansas, KY: Kentucky, LA: Louisiana, ME: Maine, MD: Maryland, MA: Massachusetts, MI: Michigan, MN: Minnesota, MS: Mississippi, MO: Missouri, MT: Montana, NE: Nebraska, NV: Nevada, NH: New Hampshire, NJ: New Jersey, NM: New Mexico, NY: New York, NC: North Carolina, ND: North Dakota, OH: Ohio, OK: Oklahoma, OR: Oregon, PA: Pennsylvania, RI: Rhode Island, SC: South Carolina, SD: South Dakota, TN: Tennessee, TX: Texas. UT: Utah. VT: Vermont, VA: Virginia, WA: Washington, WV: West Virginia, WI: Wisconsin, WY: Wyoming.
(DOCX)

S1 Table Final variables used in the study based on BRFSS codebook. (DOCX)

S2 Table Missing Data for demographic and sociographic factors. (DOCX)

S3 Table Demographic factors and days with not good mental health percentage among stroke survivors with FMD defined as ≥6 days of poor mental health in the past 30 days. (DOCX)

S4 Table Odds ratio of stroke survivor FMD, defined as ≥6 days of poor mental health in the past 30 days, in the United States. Adjusted for sociodemographic factors (age, race, sex, BMI, income, health insurance, education status).
(DOCX)
