# Supplementary material for: Prevalence of frequent mental distress and associated factors among stroke survivors
Source: PLoS One. 2026 Nov 11;21(11):e0361900. doi: 10.1371/journal.pone.0361900 (PMC13754131; doi:10.1371/journal.pone.0361900)
Supplement: Supplementary file 2 [file pone.0361900.s002.docx]

**Fig S2. Adjusted and unadjusted odds ratios of FMD, defined as ≥6 days of poor mental health in the past 30 days, among stroke and MI survivors compared to the healthy adult population.**
