# Supplementary material for: Prevalence of frequent mental distress and associated factors among stroke survivors
Source: PLoS One. 2026 Nov 11;21(11):e0361900. doi: 10.1371/journal.pone.0361900 (PMC13754131; doi:10.1371/journal.pone.0361900)
Supplement: Supplementary file 3 [file pone.0361900.s003.docx]

**Fig S3. FMD (6 or more days) percentages in stroke survivors in the United States.** AL:

Alabama, AK: Alaska, AZ: Arizona, AR: Arkansas, CA: California, CO: Colorado, CT:

Connecticut, DE: Delaware, DC: District of Columbia, FL: Florida, GA: Georgia, HI: Hawaii, ID: Idaho, IL: Illinois, IN: Indiana, IA: Iowa, KS: Kansas, KY: Kentucky, LA: Louisiana, ME:

Maine, MD: Maryland, MA: Massachusetts, MI: Michigan, MN: Minnesota, MS: Mississippi,

MO: Missouri, MT: Montana, NE: Nebraska, NV: Nevada, NH: New Hampshire, NJ: New

Jersey, NM: New Mexico, NY: New York, NC: North Carolina, ND: North Dakota, OH: Ohio, OK: Oklahoma, OR: Oregon, PA: Pennsylvania, RI: Rhode Island, SC: South Carolina, SD:

South Dakota, TN: Tennessee, TX: Texas. UT: Utah. VT: Vermont, VA: Virginia, WA:

Washington, WV: West Virginia, WI: Wisconsin, WY: Wyoming.
