# Supplementary material for: Prevalence of frequent mental distress and associated factors among stroke survivors
Source: PLoS One. 2026 Nov 11;21(11):e0361900. doi: 10.1371/journal.pone.0361900 (PMC13754131; doi:10.1371/journal.pone.0361900)
Supplement: Supplementary file 4 [file pone.0361900.s004.docx]

**Table S1. Final variables used in the study based on BRFSS codebook**

| **Variable** | **Variable Name in**  **BRFSS codebook** | **Variable Categories in BRFSS codebook** | **Re-Code Values** |
| --- | --- | --- | --- |
| Age | **_AGE65YR** | 18 to 64 | 1 |
|  |  | 65 or older | 2 |
| Race | **_RACEGR3,**  **_RACEGR4** | White only, nonHispanic | 1 |
|  |  | Black only, nonHispanic | 2 |
|  |  | Other race only, nonHispanic | 3 |
|  |  | Multiracial, nonHispanic | 4 |
|  |  | Hispanic | 5 |
| Sex | **SEX, SEX1, _SEX** | Male | 1 |
|  |  | Female | 2 |
| BMI | **_BMI5CAT** | Underweight (<18.5 kg/m | 1 |
|  |  | Normal weight (18.5–  24.9 kg/m²) | 2 |
|  |  | Overweight (25.0–  29.9 kg/m²) | 3 |
|  |  | Obese (≥30.0 kg/m²) | 4 |
| Income | **_INCOMG,**  **_INCOMG1** | Less than $15,000 | 1 |
|  |  | $15,000 to less than  $25,000 | 2 |
|  |  | $25,000 to less than  $35,000 | 3 |
|  |  | $35,000 to less than  $50, 000 | 4 |
|  |  | $50, 000 to less than  $100,000 | 5 |
|  |  | $100,000 to less than  $200,000 | 6 |
|  |  | $200,000 or more | 7 |
| Health Insurance | **HLTHPLN1,**  **_HLTHPLN,**  **_HLTHPL1** | Yes | 1 |
|  |  | No | 2 |
| Education Status | **_EDUCAG** | Did not graduate High School | 1 |
|  |  | Graduated High School | 2 |
|  |  | Attended College or Technical School | 3 |
|  |  | Graduated from  College or Technical  School | 4 |
| Metropolitan | **MSCODE** | In the center city of a  Metropolitan Status  Area (MSA) | Urban |
|  |  | Outside the center city of an MSA but inside the county containing the center city | Urban |
|  |  | Inside a suburban county of the MSA | Urban |
|  |  | Not in an MSA | Rural |
| Stroke belt residence | **StrokeBelt Derived variable for this study** | Alabama, Arkansas,  Georgia, Louisiana,  Mississippi, North  Carolina, South  Carolina, and  Tennessee | Yes |
|  |  | Other States | No |
| Depression | **ADDEPEV2,**  **ADDEPEV3** | Yes | 1 |
|  |  | No | 2 |
| Number of Days  Mental Health Not  Good | **MENTHLTH** | Continuous (1-30 days) |  |
| Mental Health Status | **_MENT14D** | Zero days when mental health not good | 1 |
|  |  | 1-13 days when mental health not good | 2 |
|  |  | 14+ days when mental health not good | 3 |
| Stroke Survivor | **CVDSTRK3** | No | 2 |
|  |  | No | 2 |
