# Supplementary material for: Prevalence of frequent mental distress and associated factors among stroke survivors
Source: PLoS One. 2026 Nov 11;21(11):e0361900. doi: 10.1371/journal.pone.0361900 (PMC13754131; doi:10.1371/journal.pone.0361900)
Supplement: Supplementary file 5 [file pone.0361900.s005.docx]

**Table S2. Missing Data for demographic and sociographic factors**

| **Factor** | **Number of nonmissing records** | **Number of missing records** |
| --- | --- | --- |
| **Age** |  |  |
| 18 to 64 | 40730 | 13271 |
| 65 or older | 64959 | 27221 |
| **Race** |  |  |
| White only, non-Hispanic | 80676 | 28721 |
| Black only, non-Hispanic | 11386 | 4723 |
| Other race only, non-Hispanic | 5193 | 1894 |
| Multiracial, non-Hispanic | 2914 | 997 |
| Hispanic | 5520 | 2285 |
| **Sex** |  |  |
| Male | 50751 | 16141 |
| Female | 54938 | 26112 |
| **BMI** |  |  |
| Underweight | 2134 | 827 |
| Normal weight | 27153 | 9689 |
| Overweight | 36795 | 10855 |
| Obese | 39607 | 10381 |
| **Income** |  |  |
| Less than $15,000 | 18414 | 2534 |
| $15,000 to less than $25,000 | 24999 | 2993 |
| $25,000 to less than $35,000 | 15328 | 1705 |
| $35,000 to less than $50, 000 | 14949 | 2084 |
| $50, 000 to less than $100,000 | 27414 | 2208 |
| $100,000 to less than $200,000 | 3687 | 303 |
| $200,000 or more | 898 | 94 |
| **Health Insurance** |  |  |
| Insured | 101238 | 37602 |
| Uninsured | 4451 | 1927 |
| **Education Status** |  |  |
| Did not graduate High School | 11231 | 6574 |
| Graduated High School | 33098 | 14164 |
| Attended College or Technical School | 32143 | 11475 |
| Graduated from College or Technical School | 29217 | 9495 |
