# Supplementary material for: Prevalence of frequent mental distress and associated factors among stroke survivors
Source: PLoS One. 2026 Nov 11;21(11):e0361900. doi: 10.1371/journal.pone.0361900 (PMC13754131; doi:10.1371/journal.pone.0361900)
Supplement: Supplementary file 6 [file pone.0361900.s006.docx]

**Table S3. Demographic factors and days with not good mental health percentage among stroke survivors with FMD defined as ≥6 days of poor mental health in the past 30 days.**

| **Factor** | **N**  **(Unweighted)** | **0 to 5 Days with Not**  **Good Mental Health**  **Percentage (95% CI)**  **(Weighted)** | **6 or More Days with**  **Not Good Mental**  **Health Percentage**  **(95% CI) (Weighted)** | ***p*-Value** |
| --- | --- | --- | --- | --- |
| **Age** |  |  |  | < 0.0001 |
| Age 18 to 64 | 52559 | 58.9 (58.0-59.8) | 41.1 (40.2-42.0) |  |
| Age 65 or older | 89177 | 80.3 (79.7-80.9) | 19.7 (19.1-20.3) |  |
| **Race** |  |  |  | < 0.0001 |
| White only, NonHispanic | 106418 | 71.9 (71.3-72.5) | 28.1 (27.5-28.7) |  |
| Black only, NonHispanic | 15507 | 69.0 (67.5-70.4) | 31.0 (29.6-32.5) |  |
| Other race only, Non-Hispanic | 6822 | 66.7 (63.6-69.8) | 33.3 (30.2-36.4) |  |
| Multiracial, NonHispanic | 3769 | 61.2 (57.6-64.8) | 38.8 (35.2-42.4) |  |
| Hispanic | 7511 | 63.6 (61.3-65.9) | 36.4 (34.1-38.7) |  |
| **Sex** |  |  |  | < 0.0001 |
| Male | 64953 | 73.5 (72.7-74.3) | 26.5 (25.7-27.3) |  |
| Female | 78441 | 66.4 (65.6-67.2) | 33.6 (32.8-34.4) |  |
| **BMI** |  |  |  | < 0.0001 |
| Underweight | 2861 | 61.5 (57.5-65.4) | 38.5 (34.6-42.5) |  |
| Normal Weight | 35616 | 71.6 (70.5-72.8) | 28.4 (27.2-29.5) |  |
| Overweight | 46300 | 73.0 (72.1-74.0) | 27.0 (26.0-27.9) |  |
| Obese | 48662 | 66.1 (65.1-67.0) | 33.9 (33.0-34.9) |  |
| **Income** |  |  |  | < 0.0001 |
| Less than $15,000 | 20175 | 54.2 (52.6-55.7) | 45.8 (44.3-47.4) |  |
| $15,000 to <  $25,000 | 27156 | 64.8 (63.5-66.1) | 35.2 (33.9-36.5) |  |
| $25,000 to <  $35,000 | 16613 | 69.8 (68.2-71.3) | 30.2 (28.7-31.8) |  |
| $35,000 to <  $50,000 | 16020 | 75.3 (73.8-76.9) | 24.7 (23.1-26.2) |  |
| $50,000 to <  $100,000 | 29194 | 79.0 (77.8-80.1) | 21.0 (19.9-22.2) |  |
| $100,000 to <  $200,000 | 3950 | 79.4 (76.0-82.7) | 20.6 (17.3-24.0) |  |
| $200,000 or more | 982 | 80.2 (74.7-85.8) | 19.8 (14.2-25.3) |  |
| **Health Insurance** |  |  |  | < 0.0001 |
| Insured | 134761 | 70.9 (70.3-71.5) | 29.1 (28.5-29.7) |  |
| Uninsured | 6141 | 56.0 (53.2-58.8) | 44.0 (41.2-46.8) |  |
| **Education Status** |  |  |  | < 0.0001 |
| Did not graduate High School | 16824 | 62.1 (60.6-63.6) | 37.9 (36.4-39.4) |  |
| Graduated High School | 45667 | 69.4 (68.4-70.4) | 30.6 (29.6-31.6) |  |
| Attended College or Technical School | 42468 | 70.4 (69.4-71.4) | 29.6 (28.6-30.6) |  |
| Graduated from  College or  Technical | 37946 | 78.8 (77.9-79.8) | 21.2 (20.2-22.1) |  |
| **Stroke Belt**  **Residence** |  |  |  | < 0.0001 |
| Non-stroke Belt | 112526 | 70.5 (69.8-71.2) | 29.5 (28.8-30.2) |  |
| Stroke Belt | 30944 | 67.9 (67.1-68.8) | 32.1 (31.2-32.9) |  |
| **Rural Status** |  |  |  | < 0.05 |
| Non-rural | 42034 | 76.0 (75.0-76.9) | 24.0 (23.1-25.0) |  |
| Rural | 23473 | 73.3 (71.6-74.9) | 26.7 (25.1-28.4) |  |
