# Supplementary material for: Prevalence of frequent mental distress and associated factors among stroke survivors
Source: PLoS One. 2026 Nov 11;21(11):e0361900. doi: 10.1371/journal.pone.0361900 (PMC13754131; doi:10.1371/journal.pone.0361900)
Supplement: Supplementary file 7 [file pone.0361900.s007.docx]

**Table S4. Odds ratio of stroke survivor FMD, defined as ≥6 days of poor mental health in the past 30 days, in the United States.** Adjusted for sociodemographic factors (age, race, sex, BMI, income, health insurance, education status).

| **Variable (Reference)** | **Unadjusted Odds Ratio (95% CI)** | ***p*-Value** | **Adjusted Odds Ratio (95% CI)** | ***p-Value*** |
| --- | --- | --- | --- | --- |
| **Age** |  |  |  |  |
| Less than 65 (reference) | - | - | - | - |
| 65 and above | 0.35 ( 0.33 - 0.38 ) | < 0.0001 | 0.49 ( 0.45 - 0.52 ) | < 0.0001 |
| **Race** |  |  |  |  |
| White (reference) | - | - | - | - |
| Black only, non-Hispanic | 1.13 ( 1.03 - 1.23 ) | < 0.05 | 0.96 ( 0.86 - 1.07 ) | 0.4971 |
| Other races only, non-Hispanic | 1.23 ( 1.04 - 1.45 ) | < 0.05 | 1.2 ( 0.99 - 1.46 ) | 0.0663 |
| Multiracial, non-Hispanic | 1.66 ( 1.4 - 1.98 ) | < 0.0001 | 1.2 ( 0.96 - 1.49 ) | 0.1047 |
| Hispanic | 1.35 ( 1.2 - 1.53 ) | < 0.0001 | 0.99 ( 0.85 - 1.15 ) | 0.8999 |
| **Sex** |  |  |  |  |
| Male (reference) | - | - | - | - |
| Female | 1.51 ( 1.43 - 1.61 ) | < 0.0001 | 1.21 ( 1.12 - 1.3 ) | < 0.0001 |
| **BMI** |  |  |  |  |
| Underweight (reference) | - | - | - | - |
| Normal Weight | 0.59 ( 0.48 - 0.72 ) | < 0.0001 | 0.72 ( 0.55 - 0.93 ) | < 0.05 |
| Overweight | 0.55 ( 0.45 - 0.67 ) | < 0.0001 | 0.69 ( 0.53 - 0.89 ) | < 0.05 |
| Obese | 0.75 ( 0.61 - 0.91 ) | < 0.05 | 0.73 ( 0.56 - 0.94 ) | < 0.05 |
| **Income** |  |  |  |  |
| Less than $15,000 (reference) | - | - | - | - |
| $15,000 to < $25,000 | 0.64 ( 0.58 - 0.7 ) | < 0.0001 | 0.82 ( 0.74 - 0.92 ) | < 0.05 |
| $25,000 to < $35,000 | 0.51 ( 0.46 - 0.57 ) | < 0.0001 | 0.75 ( 0.66 - 0.84 ) | < 0.0001 |
| $35,000 to < $50,000 | 0.38 ( 0.34 - 0.42 ) | < 0.0001 | 0.59 ( 0.52 - 0.67 ) | < 0.0001 |
| $50,000 to < $100,000 | 0.31 ( 0.28 - 0.34 ) | < 0.0001 | 0.52 ( 0.46 - 0.59 ) | < 0.0001 |
| $100,000 to < $200,000 | 0.3 ( 0.24 - 0.38 ) | < 0.0001 | 0.49 ( 0.37 - 0.65 ) | < 0.0001 |
| $200,000 or more | 0.25 ( 0.16 - 0.38 ) | < 0.0001 | 0.46 ( 0.3 - 0.73 ) | < 0.05 |
| **Health Insurance** |  |  |  |  |
| Insured (reference) | - | - | - | - |
| Uninsured | 1.96 ( 1.71 - 2.24 ) | < 0.0001 | 1.2 ( 1.01 - 1.44 ) | < 0.05 |
| **Education Status** |  |  |  |  |
| Did not graduate high school (reference) | - | - | - | - |
| Graduated high school | 0.71 ( 0.64 - 0.77 ) | < 0.0001 | 0.91 ( 0.81 - 1.03 ) | 0.1303 |
| Attended college or technical school | 0.69 ( 0.62 - 0.75 ) | < 0.0001 | 0.89 ( 0.79 - 1.01 ) | 0.0656 |
| Graduated from college or technical school | 0.42 ( 0.38 - 0.47 ) | < 0.0001 | 0.72 ( 0.63 - 0.82 ) | < 0.0001 |
