# Supplementary material for: Prevalence of frequent mental distress and associated factors among stroke survivors
Source: PLoS One. 2026 Nov 11;21(11):e0361900. doi: 10.1371/journal.pone.0361900 (PMC13754131; doi:10.1371/journal.pone.0361900)
Supplement: Supplementary file 8 [file pone.0361900.s009.docx]

**Response to Reviewers**

We thank the reviewers for their thoughtful and constructive feedback. Below, we provide point‑by‑point responses and describe the revisions made to strengthen the manuscript.

**Reviewer #1**

1. Reviewer comment: “It would be interesting for the authors to add the specific clinical data in the subgroup of very old patients (85 years or older)… (Cerebrovasc Dis 2025;54:11‑19).”

Response: We appreciate this important suggestion. Because BRFSS does not isolate the ≥85 acute stroke subgroup, we were unable to directly analyze this population. However, we have now incorporated this point into the limitation section. We added a statement noting that very old stroke patients have distinct demographic and vascular profiles, and that BRFSS cannot capture these differences. We also cite the recommended Cerebrovascular Diseases study and highlight this as a direction for future research.

2. Reviewer comment: “It would be interesting to know the frequency of cognitive impairment in the study sample…”

Response: We agree that cognitive impairment is highly relevant. Unfortunately, BRFSS did not administer the cognitive impairment question to most respondents in our sample. Of ~148,000 individuals, ~140,000 had missing data for this item, making meaningful analysis impossible. We have added this explanation to the limitations section and noted that future studies using datasets with more data are needed.

3. Reviewer comment: “The authors should clearly mention… correlation between white matter alterations, cognitive impairment, and outcome… The plausible relationship of mental distress and white matter alterations should be emphasized.”

Response: We have added a part to the discussion section addressing this point. The new text incorporates the recommended BMC Neurology reference and addresses the association of cognitive impairment driven by white matter alterations to emphasize the plausible pathway linking white matter disruption to elevated mental distress after stroke.

4. Reviewer comment: “Indicate that a limitation… would be the assessment of poor sleep quality and sleep‑related breathing disorders… (Curr Issues Mol Biol. 2025).”

Response: We have added this limitation to the limitations section. The revised text notes that BRFSS does not capture sleep quality or sleep‑related breathing disorders, despite evidence that poor sleep and obstructive sleep apnea worsen stroke outcomes. We also cite the recommended Curr Issues in Molecular Biology reference and identify this as a direction for future research.

5. Reviewer comment: “A brief concluding comment on other possible lines of future research would be appreciated.”

Response: We expanded the conclusion to include a final sentence outlining future research needs, adding on to what written previously, based on changes we made in the limitations section.

**Reviewer #2**

Reviewer comment: “Did you consider other confounding factors such as hyperlipidemia, underlying mental health disorders, use of antidepressants…?”

Response: We appreciate this thoughtful comment. We considered vascular risk factors such as hyperlipidemia; however, because hyperlipidemia is primarily a risk factor for stroke rather than a determinant of mental distress, adjusting for it would not meaningfully clarify the relationship between stroke and FMD. Our aim was to characterize the real‑world mental health burden among stroke survivors, who commonly have multiple vascular comorbidities, and excluding or adjusting for hyperlipidemia would have reduced the generalizability of our findings.

Regarding underlying mental health disorders, we did not exclude individuals with diagnosed or subclinical conditions because these fall within the definition of FMD. FMD is intended to serve as a broad screening measure that captures both diagnosed disorders and undiagnosed symptoms, allowing us to examine mental distress across the full spectrum rather than restricting the sample to only those with pre‑existing conditions.

Finally, BRFSS does not collect information on antidepressant use or adherence. We have added this point to the limitations section, noting that antidepressant use may be a significant confounder and should be incorporated into future research using clinical datasets with medication data.
