# Supplementary material for: Prevalence of frequent mental distress and associated factors among stroke survivors
Source: PLoS One. 2026 Nov 11;21(11):e0361900. doi: 10.1371/journal.pone.0361900 (PMC13754131; doi:10.1371/journal.pone.0361900)
Supplement: Supplementary file 9 [file pone.0361900.s010.docx]

**Response to Reviewers**

We thank the reviewers for their thoughtful and constructive feedback. Below, we provide point‑by‑point responses and describe the revisions made to strengthen the manuscript.

**Reviewer #1**

1. Reviewer comment: “However, reference #38 and #39 are the same reference. They must place properly in-text bibliographic reference #39: Uscamaita, K et al. Curr Issues Mol Biol. 2025, 47(3), 138.”

Response: Changed the last citation to the correct citation.
