# Supplementary material for: Prevalence of frequent mental distress and associated factors among stroke survivors
Source: PLoS One. 2026 Nov 11;21(11):e0361900. doi: 10.1371/journal.pone.0361900 (PMC13754131; doi:10.1371/journal.pone.0361900)
Supplement: Peer review history

# Peer review: Decision Letter 0

**DOI:** 10.1371/journal.pone.0361900.r001

**Authors:** Satish Patil

2 Sep 2026

Dear Dr. Zand,

Thank you for submitting your manuscript to PLOS One. After careful consideration, we feel that it has merit but does not fully meet PLOS One’s publication criteria as it currently stands. Therefore, we invite you to submit a revised version of the manuscript that addresses the points raised during the review process.

Please submit your revised manuscript by Oct 17 2026 11:59PM. If you will need more time than this to complete your revisions, please reply to this message or contact the journal office at plosone@plos.org. When you're ready to submit your revision, log on to https://www.editorialmanager.com/pone/ and select the 'Submissions Needing Revision' folder to locate your manuscript file.

- A letter that responds to each point raised by the academic editor and reviewer(s). You should upload this letter as a separate file labeled 'Response to Reviewers'.
- A marked-up copy of your manuscript that highlights changes made to the original version. You should upload this as a separate file labeled 'Revised Manuscript with Track Changes'.
- An unmarked version of your revised paper without tracked changes. You should upload this as a separate file labeled 'Manuscript'.

If you would like to make changes to your financial disclosure, please include your updated statement in your cover letter. Guidelines for resubmitting your figure files are available below the reviewer comments at the end of this letter.

If applicable, we recommend that you deposit your laboratory protocols in protocols.io to enhance the reproducibility of your results. Protocols.io assigns your protocol its own identifier (DOI) so that it can be cited independently in the future. For instructions see: https://journals.plos.org/plosone/s/submission-guidelines#loc-laboratory-protocols. Additionally, PLOS One offers an option for publishing peer-reviewed Lab Protocol articles, which describe protocols hosted on protocols.io. Read more information on sharing protocols at https://plos.org/protocols?utm_medium=editorial-email&utm_source=authorletters&utm_campaign=protocols.

As the corresponding author, your ORCID iD is verified in the submission system and will appear in the published article. PLOS supports the use of ORCID, and we encourage all coauthors to register for an ORCID iD and use it as well. Please encourage your coauthors to verify their ORCID iD within the submission system before final acceptance, as unverified ORCID iDs will not appear in the published article. *Only*  the individual author can complete the verification step; PLOS staff *cannot*  verify ORCID iDs on behalf of authors.

We look forward to receiving your revised manuscript.

Kind regards,

Satish G Patil, PhD

Academic Editor

PLOS One

Journal Requirements:

When submitting your revision, we need you to address these additional requirements.

1. Please ensure that your manuscript meets PLOS ONE's style requirements, including those for file naming. The PLOS ONE style templates can be found at

https://journals.plos.org/plosone/s/file?id=wjVg/PLOSOne_formatting_sample_main_body.pdf and

https://journals.plos.org/plosone/s/file?id=ba62/PLOSOne_formatting_sample_title_authors_affiliations.pdf

2. We note that Figures 2 and 3 in your submission contain map/satellite images which may be copyrighted. All PLOS content is published under the Creative Commons Attribution License (CC BY 4.0), which means that the manuscript, images, and Supporting Information files will be freely available online, and any third party is permitted to access, download, copy, distribute, and use these materials in any way, even commercially, with proper attribution. For these reasons, we cannot publish previously copyrighted maps or satellite images created using proprietary data, such as Google software (Google Maps, Street View, and Earth). For more information, see our copyright guidelines: http://journals.plos.org/plosone/s/licenses-and-copyright.

We require you to either (1) present written permission from the copyright holder to publish these figures specifically under the CC BY 4.0 license, or (2) remove the figures from your submission:

a. You may seek permission from the original copyright holder of Figures 2 and S3 to publish the content specifically under the CC BY 4.0 license.

We recommend that you contact the original copyright holder with the Content Permission Form (http://journals.plos.org/plosone/s/file?id=7c09/content-permission-form.pdf) and the following text:

“I request permission for the open-access journal PLOS ONE to publish XXX under the Creative Commons Attribution License (CCAL) CC BY 4.0 (http://creativecommons.org/licenses/by/4.0/). Please be aware that this license allows unrestricted use and distribution, even commercially, by third parties. Please reply and provide explicit written permission to publish XXX under a CC BY license and complete the attached form.”

Please upload the completed Content Permission Form or other proof of granted permissions as an "Other" file with your submission.

In the figure caption of the copyrighted figure, please include the following text: “Reprinted from [ref] under a CC BY license, with permission from [name of publisher], original copyright [original copyright year].”

b. If you are unable to obtain permission from the original copyright holder to publish these figures under the CC BY 4.0 license or if the copyright holder’s requirements are incompatible with the CC BY 4.0 license, please either i) remove the figure or ii) supply a replacement figure that complies with the CC BY 4.0 license. Please check copyright information on all replacement figures and update the figure caption with source information. If applicable, please specify in the figure caption text when a figure is similar but not identical to the original image and is therefore for illustrative purposes only.

The following resources for replacing copyrighted map figures may be helpful:

USGS National Map Viewer (public domain): http://viewer.nationalmap.gov/viewer/

The Gateway to Astronaut Photography of Earth (public domain): http://eol.jsc.nasa.gov/sseop/clickmap/

Maps at the CIA (public domain): https://www.cia.gov/library/publications/the-world-factbook/index.html and https://www.cia.gov/library/publications/cia-maps-publications/index.html

NASA Earth Observatory (public domain): http://earthobservatory.nasa.gov/

Landsat: http://landsat.visibleearth.nasa.gov/

USGS EROS (Earth Resources Observatory and Science (EROS) Center) (public domain): http://eros.usgs.gov/#

Natural Earth (public domain): http://www.naturalearthdata.com/

3. If the reviewer comments include a recommendation to cite specific previously published works, please review and evaluate these publications to determine whether they are relevant and should be cited. There is no requirement to cite these works unless the editor has indicated otherwise.

[Note: HTML markup is below. Please do not edit.]

Reviewers' comments:

Reviewer's Responses to Questions

**Comments to the Author**

1. Is the manuscript technically sound, and do the data support the conclusions?

Reviewer #1: Yes

Reviewer #2: Yes

**********

2. Has the statistical analysis been performed appropriately and rigorously?

Reviewer #1: Yes

Reviewer #2: Yes

**********

3. Have the authors made all data underlying the findings in their manuscript fully available?

The PLOS Data policy

Reviewer #1: Yes

Reviewer #2: Yes

**********

4. Is the manuscript presented in an intelligible fashion and written in standard English?

Reviewer #1: Yes

Reviewer #2: Yes

**********

Reviewer #1: The authors present the results of a cross-sectional analysis using 8 years of data (2016 to 2023) from the nationally representative Behavioral Risk Factor Surveillance System (BRFSS) to analyze the prevalence and associated factors of frequent mental distress (FMD) among U.S. stroke survivors. The authors suggest that FMD is more prevalent in younger adults, women, Hispanics, those with lower income, lower education status, the uninsured, and stroke survivors residing in the Stroke belt and Appalachian states. FMD is also more prevalent among stroke survivors compared to individuals with MI or healthy adults. The study is potentially interesting, but can be improved if the following considerations are addressed:

1.It would be interesting for the authors to add the specific clinical data in the subgroup of very old patients (85 years or older) with acute stroke, since the demographics and risk factors are quite different in this age segment of stroke patients (see data and comment on the study published in Cerebrovasc Dis 2025;54(1):11-19. doi: 10.1159/000536436. Epub 2024 Jan 29. PMID: 38286114).

2.It would be interesting to know the frequency of cognitive impairment in the study sample, and in the different subgroups analyzed.

3.The authors should clearly mention in the Discussion section that there is a significant correlation between white matter alterations, cognitive impairment, and outcome for example in metabolic syndrome patients. There is a cluster of silent vascular risk factors that may influence cerebrovascular pathology during the aging of patients (see data and comment on the study published in BMC Neurol. 2010 Jul 27;10:64. doi: 10.1186/1471-2377-10-64. PMID: 20663196; PMCID: PMC2920865). The plausible relationship of mental distress and white matter alterations should be emphasized.

4.The authors should indicate that a limitation of the study and an essential line of future research would be precisely the assessment of the role of poor sleep quality and the effect of “sleep-related breathing disorders”, anxiety and depression symptoms in ischemic stroke outcome (see and add this reference: Curr Issues Mol Biol. 2025, 47(3), 138; https://doi.org/10.3390/cimb47030138).

5.A brief concluding comment on other possible lines of future research on the presented topic would be appreciated

Reviewer #2: this paper looks into the association between frequent mental distress and stroke, it also aimed to look into associated sociodemographic and the presence of FMD in those with stroke vs MI. The objectives have been well addressed how ever did you consider other confounding factors such as: excluding people who reported hyperlipidemia, presence of underlying mental health disorders (subclinical or full blown mood disorders, use of antidepressants..) ?

**********

what does this mean?). If published, this will include your full peer review and any attached files.

If you choose “no”, your identity will remain anonymous but your review may still be made public.

**Do you want your identity to be public for this peer review?**  For information about this choice, including consent withdrawal, please see our Privacy Policy

Reviewer #1: **Yes:**  Adrià Arboix

Reviewer #2: No

**********

[NOTE: If reviewer comments were submitted as an attachment file, they will be attached to this email and accessible via the submission site. Please log into your account, locate the manuscript record, and check for the action link "View Attachments". If this link does not appear, there are no attachment files.]

To ensure your figures meet our technical requirements, please review our figure guidelines: https://journals.plos.org/plosone/s/figures

You may also use PLOS’s free figure tool, NAAS, to help you prepare publication quality figures: https://journals.plos.org/plosone/s/figures#loc-tools-for-figure-preparation.

NAAS will assess whether your figures meet our technical requirements by comparing each figure against our figure specifications.

# Author comment: Author response to Decision Letter 1

**DOI:** 10.1371/journal.pone.0361900.r002

14 Sep 2026

Reviewer #1

1. Reviewer comment: “It would be interesting for the authors to add the specific clinical data in the subgroup of very old patients (85 years or older)… (Cerebrovasc Dis 2025;54:11 19).”

Response: We appreciate this important suggestion. Because BRFSS does not isolate the ≥85 acute stroke subgroup, we were unable to directly analyze this population. However, we have now incorporated this point into the limitation section. We added a statement noting that very old stroke patients have distinct demographic and vascular profiles, and that BRFSS cannot capture these differences. We also cite the recommended Cerebrovascular Diseases study and highlight this as a direction for future research.

2. Reviewer comment: “It would be interesting to know the frequency of cognitive impairment in the study sample…”

Response: We agree that cognitive impairment is highly relevant. Unfortunately, BRFSS did not administer the cognitive impairment question to most respondents in our sample. Of ~148,000 individuals, ~140,000 had missing data for this item, making meaningful analysis impossible. We have added this explanation to the limitations section and noted that future studies using datasets with more data are needed.

3. Reviewer comment: “The authors should clearly mention… correlation between white matter alterations, cognitive impairment, and outcome… The plausible relationship of mental distress and white matter alterations should be emphasized.”

Response: We have added a part to the discussion section addressing this point. The new text incorporates the recommended BMC Neurology reference and addresses the association of cognitive impairment driven by white matter alterations to emphasize the plausible pathway linking white matter disruption to elevated mental distress after stroke.

4. Reviewer comment: “Indicate that a limitation… would be the assessment of poor sleep quality and sleep related breathing disorders… (Curr Issues Mol Biol. 2025).”

Response: We have added this limitation to the limitations section. The revised text notes that BRFSS does not capture sleep quality or sleep related breathing disorders, despite evidence that poor sleep and obstructive sleep apnea worsen stroke outcomes. We also cite the recommended Curr Issues in Molecular Biology reference and identify this as a direction for future research.

5. Reviewer comment: “A brief concluding comment on other possible lines of future research would be appreciated.”

Response: We expanded the conclusion to include a final sentence outlining future research needs, adding on to what written previously, based on changes we made in the limitations section.

Reviewer #2

Reviewer comment: “Did you consider other confounding factors such as hyperlipidemia, underlying mental health disorders, use of antidepressants…?”

Response: We appreciate this thoughtful comment. We considered vascular risk factors such as hyperlipidemia; however, because hyperlipidemia is primarily a risk factor for stroke rather than a determinant of mental distress, adjusting for it would not meaningfully clarify the relationship between stroke and FMD. Our aim was to characterize the real‑world mental health burden among stroke survivors, who commonly have multiple vascular comorbidities, and excluding or adjusting for hyperlipidemia would have reduced the generalizability of our findings.

Regarding underlying mental health disorders, we did not exclude individuals with diagnosed or subclinical conditions because these fall within the definition of FMD. FMD is intended to serve as a broad screening measure that captures both diagnosed disorders and undiagnosed symptoms, allowing us to examine mental distress across the full spectrum rather than restricting the sample to only those with pre‑existing conditions.

Finally, BRFSS does not collect information on antidepressant use or adherence. We have added this point to the limitations section, noting that antidepressant use may be a significant confounder and should be incorporated into future research using clinical datasets with medication data.

Attachment Submitted filename: Response to Reviewers.docx

# Peer review: Decision Letter 1

**DOI:** 10.1371/journal.pone.0361900.r003

**Authors:** Satish Patil

24 Sep 2026

Dear Dr. Zand,

Thank you for submitting your manuscript to PLOS One. After careful consideration, we feel that it has merit but does not fully meet PLOS One’s publication criteria as it currently stands. Therefore, we invite you to submit a revised version of the manuscript that addresses the points raised during the review process.

plosone@plos.org. When you're ready to submit your revision, log on to https://www.editorialmanager.com/pone/ and select the 'Submissions Needing Revision' folder to locate your manuscript file.

- A letter that responds to each point raised by the academic editor and reviewer(s). You should upload this letter as a separate file labeled 'Response to Reviewers'.
- A marked-up copy of your manuscript that highlights changes made to the original version. You should upload this as a separate file labeled 'Revised Manuscript with Track Changes'.
- An unmarked version of your revised paper without tracked changes. You should upload this as a separate file labeled 'Manuscript'.

If you would like to make changes to your financial disclosure, please include your updated statement in your cover letter. Guidelines for resubmitting your figure files are available below the reviewer comments at the end of this letter.

If applicable, we recommend that you deposit your laboratory protocols in protocols.io to enhance the reproducibility of your results. Protocols.io assigns your protocol its own identifier (DOI) so that it can be cited independently in the future. For instructions see: https://journals.plos.org/plosone/s/submission-guidelines#loc-laboratory-protocols. Additionally, PLOS One offers an option for publishing peer-reviewed Lab Protocol articles, which describe protocols hosted on protocols.io. Read more information on sharing protocols at https://plos.org/protocols?utm_medium=editorial-email&utm_source=authorletters&utm_campaign=protocols.

As the corresponding author, your ORCID iD is verified in the submission system and will appear in the published article. PLOS supports the use of ORCID, and we encourage all coauthors to register for an ORCID iD and use it as well. Please encourage your coauthors to verify their ORCID iD within the submission system before final acceptance, as unverified ORCID iDs will not appear in the published article. *Only*  the individual author can complete the verification step; PLOS staff *cannot*  verify ORCID iDs on behalf of authors.

We look forward to receiving your revised manuscript.

Kind regards,

Satish G Patil, PhD

Academic Editor

PLOS One

Journal Requirements:

1. If the reviewer comments include a recommendation to cite specific previously published works, please review and evaluate these publications to determine whether they are relevant and should be cited. There is no requirement to cite these works unless the editor has indicated otherwise.

2. Please review your reference list to ensure that it is complete and correct. If you have cited papers that have been retracted, please include the rationale for doing so in the manuscript text, or remove these references and replace them with relevant current references. Any changes to the reference list should be mentioned in the rebuttal letter that accompanies your revised manuscript. If you need to cite a retracted article, indicate the article’s retracted status in the References list and also include a citation and full reference for the retraction notice.

[Note: HTML markup is below. Please do not edit.]

Reviewers' comments:

Reviewer's Responses to Questions

**Comments to the Author**

Reviewer #1: All comments have been addressed

**********

2. Is the manuscript technically sound, and do the data support the conclusions?

Reviewer #1: Yes

**********

3. Has the statistical analysis been performed appropriately and rigorously?

Reviewer #1: Yes

**********

4. Have the authors made all data underlying the findings in their manuscript fully available?

The PLOS Data policy

Reviewer #1: Yes

**********

5. Is the manuscript presented in an intelligible fashion and written in standard English?

Reviewer #1: Yes

**********

Reviewer #1: The authors have made the previously recommended changes and responded the criticisms. Thank you for making these changes. However, reference #38 and #39 are the same reference. They must place properly in-text bibliographic reference #39:

Uscamaita, K et al. Curr Issues Mol Biol. 2025, 47(3), 138; https://doi.org/10.3390/cimb47030138

**********

what does this mean?). If published, this will include your full peer review and any attached files.

If you choose “no”, your identity will remain anonymous but your review may still be made public.

**Do you want your identity to be public for this peer review?**  For information about this choice, including consent withdrawal, please see our Privacy Policy

Reviewer #1: **Yes:**  Adrià Arboix

**********

[NOTE: If reviewer comments were submitted as an attachment file, they will be attached to this email and accessible via the submission site. Please log into your account, locate the manuscript record, and check for the action link "View Attachments". If this link does not appear, there are no attachment files.]

To ensure your figures meet our technical requirements, please review our figure guidelines: https://journals.plos.org/plosone/s/figures

You may also use PLOS’s free figure tool, NAAS, to help you prepare publication quality figures: https://journals.plos.org/plosone/s/figures#loc-tools-for-figure-preparation.

NAAS will assess whether your figures meet our technical requirements by comparing each figure against our figure specifications.

# Author comment: Author response to Decision Letter 2

**DOI:** 10.1371/journal.pone.0361900.r004

25 Sep 2026

Changed citation #39 to the correct citation in the references section.

Attachment Submitted filename: Response to Reviewers 2.docx

# Peer review: Decision Letter 2

**DOI:** 10.1371/journal.pone.0361900.r005

**Authors:** Satish Patil

19 Oct 2026

Prevalence of frequent mental distress and associated factors among stroke survivors

PONE-D-25-66180R2

Dear Dr. Zand,

We’re pleased to inform you that your manuscript has been judged scientifically suitable for publication and will be formally accepted for publication once it meets all outstanding technical requirements.

Within one week, you’ll receive an e-mail detailing the required amendments. When these have been addressed, you’ll receive a formal acceptance letter and your manuscript will be scheduled for publication.

An invoice will be generated when your article is formally accepted. Please note, if your institution has a publishing partnership with PLOS and your article meets the relevant criteria, all or part of your publication costs will be covered. Please make sure your user information is up-to-date by logging into Editorial Manager at Editorial Manager® and clicking the ‘Update My Information' link at the top of the page. For questions related to billing, please contact billing support.

If your institution or institutions have a press office, please notify them about your upcoming paper to help maximize its impact. If they’ll be preparing press materials, please inform our press team as soon as possible -- no later than 48 hours after receiving the formal acceptance. Your manuscript will remain under strict press embargo until 2 pm Eastern Time on the date of publication. For more information, please contact onepress@plos.org.

Kind regards,

Satish G Patil, PhD

Academic Editor

PLOS One

Additional Editor Comments (optional):

Reviewers' comments:

Reviewer's Responses to Questions

**Comments to the Author**

Reviewer #1: All comments have been addressed

**********

2. Is the manuscript technically sound, and do the data support the conclusions?

Reviewer #1: Yes

**********

3. Has the statistical analysis been performed appropriately and rigorously?

Reviewer #1: Yes

**********

4. Have the authors made all data underlying the findings in their manuscript fully available?

The PLOS Data policy

Reviewer #1: Yes

**********

5. Is the manuscript presented in an intelligible fashion and written in standard English?

Reviewer #1: Yes

**********

Reviewer #1: I am satisfied with the changes made. My sincere congratulations to the authors for their excellent work

**********

what does this mean?). If published, this will include your full peer review and any attached files.

If you choose “no”, your identity will remain anonymous but your review may still be made public.

**Do you want your identity to be public for this peer review?**  For information about this choice, including consent withdrawal, please see our Privacy Policy

Reviewer #1: **Yes:**  Adrià Arboix

**********

# Editor report: Acceptance letter

**DOI:** 10.1371/journal.pone.0361900.r006

**Authors:** Satish Patil

PONE-D-25-66180R2

PLOS One

Dear Dr. Zand,

I'm pleased to inform you that your manuscript has been deemed suitable for publication in PLOS One. Congratulations! Your manuscript is now being handed over to our production team.

At this stage, our production department will prepare your paper for publication. This includes ensuring the following:

* All references, tables, and figures are properly cited

* All relevant supporting information is included in the manuscript submission,

* There are no issues that prevent the paper from being properly typeset

You will receive further instructions from the production team, including instructions on how to review your proof when it is ready. Please keep in mind that we are working through a large volume of accepted articles, so please give us a few days to review your paper and let you know the next and final steps.

Lastly, if your institution or institutions have a press office, please let them know about your upcoming paper now to help maximize its impact. If they'll be preparing press materials, please inform our press team within the next 48 hours. Your manuscript will remain under strict press embargo until 2 pm Eastern Time on the date of publication. For more information, please contact onepress@plos.org.

You will receive an invoice from PLOS for your publication fee after your manuscript has reached the completed accept phase. If you receive an email requesting payment before acceptance or for any other service, this may be a phishing scheme. Learn how to identify phishing emails and protect your accounts at https://explore.plos.org/phishing.

If we can help with anything else, please email us at customercare@plos.org.

Thank you for submitting your work to PLOS One and supporting open access.

Kind regards,

PLOS One Editorial Office Staff

on behalf of

Prof. Dr. Satish G Patil

Academic Editor

PLOS One
